# Feeling excluded no matter what? Bias in the processing of social participation in borderline personality disorder

**DOI:** 10.1016/j.nicl.2018.04.031

**Published:** 2018-04-26

**Authors:** Anna Weinbrecht, Michael Niedeggen, Stefan Roepke, Babette Renneberg

**Affiliations:** a Clinical Psychology and Psychotherapy, Freie Universität Berlin, Berlin, Germany; b Experimental Psychology and Neuropsychology, Freie Universität Berlin, Berlin, Germany; c Department of Psychiatry and Psychotherapy, Charité – Universitätsmedizin Berlin, corporate member of Freie Universität Berlin, Humboldt-Universität zu Berlin, and Berlin Institute of Health, Campus Benjamin Franklin, Germany

**Keywords:** Borderline personality disorder, Social anxiety disorder, Social cognition, Social participation, Overinclusion, Event-related brain potentials

## Abstract

**Background:**

Patients with Borderline Personality Disorder (BPD) feel ostracized even when they are included. This might be due to a biased processing of social participation in BPD. We examined whether patients with BPD also process social overinclusion in a biased manner, i.e., whether they feel ostracized even when the degree of social participation is increased.

**Methods:**

An EEG-compatible version of Cyberball was used to investigate the effects of inclusion and overinclusion (33% vs. 45% ball receipt) on perceived ostracism, need threat and P3 amplitude, an EEG indicator for expectancy violation. Twenty-nine patients with BPD, 28 patients with Social Anxiety Disorder (SAD) and 28 healthy controls (HC) participated.

**Results:**

The P3 amplitude was enhanced for patients with BPD and SAD compared to HCs independent of condition. Both patient groups reported more perceived ostracism relative to HCs in the inclusion but not in the overinclusion condition. Only patients with BPD reported stronger need threat in both conditions.

**Conclusions:**

The EEG results imply that being socially included violates the expectations of patients with BPD, irrespective of the actual degree of social participation. However, when overincluded, patients with BPD no longer feel ostracized. Except for need threat, patients with SAD might show a comparable bias in the processing of social participation as patients with BPD.

## Highlights

• In social situations, patients with Borderline Personality Disorder generally expect to be excluded.
• This is indicated by their neurophysiological response (P3, EEG).
• Our data provide preliminary evidence for a similar bias in Social Anxiety Disorder.

## 1 Introduction

Long-lasting interpersonal problems are a hallmark feature of Borderline Personality Disorder (BPD) (American Psychiatric Association, 2013). The Cognitive Theory of Personality Disorders postulates that these interpersonal problems are related to maladaptive schemas, which lead to biases in the processing of social information (Beck et al., 2015). For example, an individual with BPD typically thinks, “I am unacceptable and others will abandon me” (Arntz, 2004; Renneberg et al., 2005). Due to this maladaptive schema and the associated processing bias, the individual may incorrectly interpret that he/she is being excluded from a group. This, in turn, may lead to interpersonal problems, e.g., impulsively insulting others, which might foster actual exclusion from social groups.

Previous research showed a characteristic bias of social information processing in BPD (e.g., Chechko et al., 2016; Niedtfeld et al., 2016) and supported the idea that the biases are related to interpersonal problems in BPD (e.g., Herbort et al., 2016; Whalley et al., 2015). Examples for the characteristic bias are that patients tend to perceive ambiguous facial expressions in a negative way (see Domes et al., 2009 for a review) and quickly feel rejected (e.g., Arntz et al., 2011; Staebler et al., 2011a). In this study, we focused on a possible bias in the processing of social participation. Most research examining social participation has relied on the Cyberball paradigm (Williams and Jarvis, 2006). Cyberball is a virtual ball-tossing game, in which the participant believes that he/she is tossing the ball with two other co-players, which are, in fact, computer-generated. Cyberball can be reliably used to induce different degrees of social participation depending on the percentage of ball tosses received (Hartgerink et al., 2015); that is, social exclusion, inclusion, and overinclusion.

According to the Cognitive Theory of Personality Disorders (Beck et al., 2015), the aforementioned biases in the processing of social information should be most prominent in *ambiguous* social situations. Being included is an ambiguous situation and leaves space for biased interpretations. Being excluded and being overincluded, by contrast, mean getting the ball almost never or almost all of the time during the Cyberball game, and cannot be seen as ambiguous. Following this line of thought, we argue that individuals with BPD should process social inclusion in a biased manner, but should show no bias in the processing of social exclusion and overinclusion.

### 1.1 Bias in the processing of social inclusion in BPD

Results of previous Cyberball studies indicated that patients with BPD process social inclusion in a biased manner: Compared to healthy controls (HCs), they estimated that the co-players tossed the ball less often to them than to the other player (Gutz et al., 2015; Renneberg et al., 2012; Staebler et al., 2011b), and they reported feeling more ostracized (Domsalla et al., 2014; Gutz et al., 2015).

Gutz et al. (2015) used an EEG-compatible version of the Cyberball game to examine an EEG correlate associated with the processing of social participation, the event-related potential P3. The P3 amplitude has mostly been studied in the oddball paradigm and peaks parietally approximately 350 ms after stimulus onset. It is inversely related to the subjective target probability (see Polich, 2007 for a review). In the social context of the Cyberball paradigm, the P3 amplitude additionally depends on the participant's prior expectation of her/his social involvement: The P3 amplitude increases when the participant's expectation of her/his degree of social participation is violated (Gutz et al., 2011; Weschke and Niedeggen, 2015). Consequently, in healthy participants, the P3 amplitude is reduced when they are included compared to when they are excluded (Gutz et al., 2011). Moreover, Niedeggen et al. (2014) showed that the P3 amplitude is enhanced when healthy participants are included compared to overincluded, indicating that overinclusion does not violate the expectations of HCs. The expectancy-based effect on the P3 amplitude has been confirmed in numerous Cyberball studies (Hajcak et al., 2005; Kiat et al., 2017; Niedeggen et al., 2017). For example, individuals who expected to be excluded because of a stereotyped cue (Kiat et al., 2017) or an inferior position (Niedeggen et al., 2017) showed a reduced P3 amplitude when excluded.

The study by Gutz et al. (2015) provided a clinical validation of the expectancy-based account: BPD patients showed an increased P3 amplitude compared to HCs in the inclusion condition. This suggests that patients with BPD expect to be excluded, and being socially included violates this expectation. Interestingly, patients with Social Anxiety Disorder (SAD) did not show an increased P3 amplitude compared to HCs. This might indicate that specifically patients with BPD experience social inclusion as an expectancy violation. However, this non-significant finding has to be interpreted with caution, because the lack of a statistically significant difference between BPD and SAD patients might also be due to low statistical power.

### 1.2 Bias in the processing of social exclusion and overinclusion in BPD

Gutz et al. (2015) compared the processing of social inclusion to the processing of social exclusion in BPD in a within-subject design: All participants were first included and then excluded. Interestingly, differences between BPD patients and HCs were mostly found for the processing of inclusion, and not for the processing of exclusion. This is in line with previous Cyberball studies (Domsalla et al., 2014; Staebler et al., 2011b) and supports the assumption that biases in the processing of social participation are most prominent in ambiguous social situations.

To our knowledge, only one study has looked at overinclusion in patients with BPD (De Panfilis et al., 2015). De Panfilis et al. (2015) compared self-reported reactions of patients with BPD to overinclusion, inclusion and exclusion in a between-subject design. The results indicated that patients with BPD and HCs do not differ in their emotional reaction to overinclusion: Patients with BPD did not report greater levels of rejection-related negative emotions than HCs when overincluded. Again, however, the lack of a difference between BPD patients and HCs might also be due to low statistical power. In contrast to the assumption that differences between patients with BPD and HCs should be most prominent in the ambiguous situation of social inclusion, patients with BPD felt less connected to their co-players in all three conditions (irrespective of the degree of social participation).

To summarize, there is sound evidence that patients with BPD process social participation in a biased manner. However, most previous studies focused on social inclusion and exclusion (Domsalla et al., 2014; Gutz et al., 2015; Renneberg et al., 2012; Staebler et al., 2011b). To our knowledge, this is the first study that applies not only self-report measures but also EEG correlates to assess the processing of social inclusion and overinclusion in BPD. More precisely, we examined the effects of inclusion and overinclusion on the P3 complex. The P3 offers an objective and continuous assessment of social expectancy violation. Moreover, EEG data present the possibility to gain a high temporal resolution, and enable a distinction between specific stages of social information processing (Bartholow and Dickter, 2007).

The primary aim was to examine whether the biased processing in BPD is specific to the ambiguous situation of social inclusion or whether patients with BPD also process overinclusion in a biased manner. Relying on the Cognitive Theory of Personality Disorders (Beck et al., 2015), we hypothesized that the P3 complex, our main outcome variable, would be enhanced for patients with BPD in the inclusion condition but not in the overinclusion condition (interaction effect) compared to HCs. A further aim of this study was to replicate the findings of Gutz et al. (2015) that patients with BPD process social inclusion in a biased manner. We hypothesized that the P3 complex would be enhanced in patients with BPD compared to HCs in the inclusion condition. In line with the study by Gutz et al. (2015), we included SAD patients as a clinical control group in order to examine whether the bias is disorder-specific.

## 2 Material and methods

### 2.1 Participants

Overall, 85 participants took part in the study: 28 HCs, 28 SAD patients and 29 BPD patients. All HCs, 11 BPD patients and 12 SAD patients were recruited via media advertisements. The remaining 18 BPD patients were recruited at the Department of Psychiatry (Charité Berlin) and the remaining 16 SAD patients were recruited at two university outpatient departments in Berlin. Outpatients and HCs were reimbursed for their participation (30 €). The 18 BPD inpatients from the Department of Psychiatry did not receive financial compensation for their participation. The study was approved by the ethics committee of the Freie Universität Berlin (ID 97 II/2016). Participation was voluntary.

Table 1 displays sociodemographic data of the sample as well as comorbid diagnoses of the BPD and SAD groups. All three groups were matched according to age, IQ and gender (all *p* > 0.6). Inclusion criteria for all participants were age between 18 and 40 years and the absence of mental retardation, epilepsy or organic brain disease. Exclusion criteria for the patients were any psychotic disorder, current substance abuse/dependency and intake of psychotropic medication within the last 4 weeks (intake of an antidepressant without any changes in dosage in the last 4 weeks was allowed).

**Table 1:** Sociodemographic characteristics and comorbid DSM-IV diagnoses.

	Descriptive statistics	HC (*n* = 28)	SAD (*n* = 28)	BPD (*n* = 29)
Gender: female	*n* (%)	24 (85)	22 (79)	25 (86)
Family status: in a relationship	*n* (%)	15 (54)	18 (64)	11 (38)
Antidepressant medication	*n* (%)	0	7 (25)	10 (35)
Age	*M* (*SD*)	28.21 (5.81)	28.86 (6.21)	27.86 (5)
IQ	*M* (*SD*)	113.71 (11.47)	114.79 (13.54)	111.47 (12.68)
Number comorbid diagnoses	*M* (*SD*)	0	1.21 (1.17)	1.69 (1.17)
MDE current (mild)	*n* (%)	0	6 (21)	2 (7)
MDE lifetime	*n* (%)	0	8 (29)	14 (48)
Any anxiety disorder except SAD	*n* (%)	0	5 (18)	16 (55)
SAD	*n* (%)	0	28 (100)	1 (4)
AVPD	*n* (%)	0	7 (25)	0
BPD	*n* (%)	0	0	29 (100)
Any other PD	*n* (%)	0	1 (4)	2 (7)

*Note*. HC = Healthy Controls, SAD = Social Anxiety Disorder, BPD = Borderline Personality Disorder; MDE = Major Depressive Episode, AVPD = Avoidant Personality Disorder, PD = Personality Disorder.

We confirmed DSM-IV diagnoses using the German versions (Wittchen et al., 1997) of the SCID I and SCID II (First et al., 1997). All interviewers were clinical psychologists, who were trained and supervised in the application of the SCID I and SCID II. Participants recruited via media advertisements were initially screened by telephone, before undergoing the clinical interview in the lab directly before the experiment. Participants recruited at the Department of Psychiatry or the university outpatient departments were interviewed at the respective treatment site.

### 2.2 Materials

#### 2.2.1 Experimental manipulation of social inclusion and overinclusion: Cyberball

**Fig. 1:**
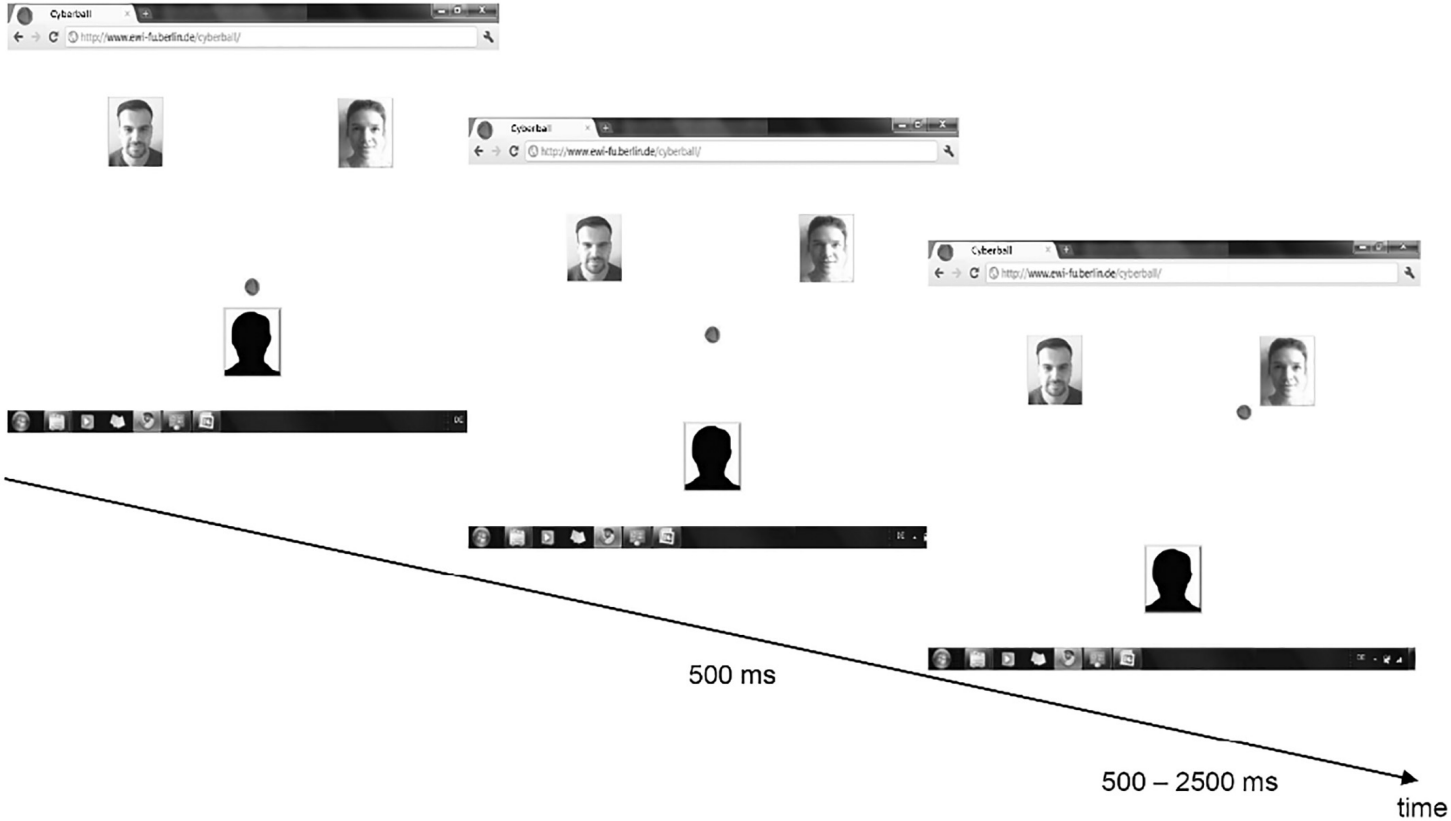
Display and sequence of the Cyberball game. The display imitated an Internet screen including the photos of two putatively human co-players. A sequence of the participant passing the ball to the right co-player is shown by way of example. To indicate the participant's ball possession, the ball appeared in front of the avatar. The participant decided to pass the ball to the right co-player by pressing a corresponding button. The ball was then displayed at a central position for 500 ms. To indicate the co-player's ball possession, the ball appeared next to the co-player for 500–2500 ms (to support the cover story of playing with humans).

To manipulate social inclusion and overinclusion, we used the EEG-compatible version (Gutz et al., 2011) of Cyberball (Williams and Jarvis, 2006). The participants believed that they were tossing a ball with two other co-players via an Internet connection (see Fig. 1). However, their co-players were computer-generated. After the experiment, participants had to rate the extent to which they believed in the cover story (“I played with the co-players via the Internet”; 1 = *not at all* to 5 = *very much*). Results indicated that participants tended to believe the cover story (*M* = 2.67, *SD* = 1.16).

To induce inclusion, each player received the ball equally often (i.e. the participant got the ball in 33% of the throws). To induce overinclusion, the participant received the ball in 45% of all throws (i.e. the co-players rarely passed the ball to each other). The participants were told a cover story that the study was aiming to examine visual mentalization capabilities, and were thus instructed to mentally visualize the ball-tossing procedure throughout the Cyberball game. After completing the experiment, all participants were debriefed.

The participant sat in front of a computer screen (7° × 7° at a viewing distance of 140 cm) on which the participant and the putatively connected co-players were displayed. Fig. 1 depicts, by way of example, the sequence of a participant passing the ball to the co-player on the top right. The Cyberball game was programmed in MATLAB (R2012a, The MathWorks, Inc.).

#### 2.2.2 Self-report measures: need threat questionnaire (NTQ; Williams et al., 2000)

We assessed need threat via the German version of the NTQ (Grzyb, 2005). The NTQ assesses four fundamental social needs that are threatened by social exclusion (Williams, 2007): belonging (e.g., “I felt disconnected”), self-esteem (e.g., “I felt good about myself”), meaningful existence (e.g., “I felt invisible”), and control (e.g., “I felt powerful”). For all four fundamental needs, three items have to be answered on a 5-point Likert scale (ranging from 1 = *not at all* to 5 = *very much*). Like Gutz et al. (2015), we used the sum score of all four subscales as a measure of total need threat (range 4–20). Higher values indicate more need threat (items were reverse-coded if necessary).

Moreover, the NTQ also assesses ostracism intensity, negative mood (e.g., “I felt sad”; range 1–4) and the estimated percentage of ball tosses received (“Assuming that the ball should be thrown to each person equally (33%), what percentage of the throws was directed to you?”). Ostracism intensity was measured by creating the sum score of two items (“I was ignored” and “I was excluded”), which were answered on the 5-point Likert scale described above (range 2–10).

### 2.3 Procedure

Prior to the lab session, participants completed a web-based battery of questionnaires. At the lab, electrodes were attached and participants completed further questionnaires, specifically the LPS-4 to measure IQ (Horn, 1962) and the Vividness of Visual Imagery Questionnaire to support the cover story (Marks, 1973). Before the EEG recording started, participants received instructions on the Cyberball game and completed a training trial. The Cyberball game consisted of two blocks: First, all participants were included (33% ball possession) and then all participants were overincluded (45% ball possession). Each block consisted of 200 throws and lasted for about 7 min. At the end of the Cyberball game, participants completed the NTQ for both conditions and were debriefed. At the beginning and end of the lab session, participants signed informed consent forms.

This study was part of a larger project. Here, we only report the part relevant to a bias in the processing of social participation. Results referring to the hypothesis of impaired positivity in SAD will be reported elsewhere.

### 2.4 EEG recording and data preparation

We recorded EEG data at frontal, central and parietal positions with Ag/AgCl electrodes. We embedded the electrodes in an electrode cap (EASYCAP, Herrsching, Germany) and filled them with electrode cream (Abralyt 2000, EASYCAP). Electrodes attached at the earlobes served as the reference (impedance for all EEG electrodes: <10 kΩ). In addition, vertical and horizontal electrooculogram were recorded to control for ocular artifacts (impedance <20 kΩ). EEG data were band-pass filtered online (0.1–200 Hz) and sampled at 500 Hz.

“Brain Vision Analyzer” was used to analyze EEG data offline (Version 2, Brain Products GmbH, Gilching, Germany). Two discrete events on the screen were of interest: (1) participant receives the ball (“self”), (2) co-player receives the ball from the other co-player (“others”). For each ball reception event, a trigger was provided and EEG was segmented accordingly (−200 to 600 ms epoch length). These EEG segments were baseline corrected (−150 to 50 ms) and filtered (0.3–30 Hz and 50 Hz). Subsequently, EEG segments with muscular or ocular artifacts as well as high Alpha activity were removed manually. A minimum number of 15 segments per event “self inclusion” and per event “self overinclusion” was defined to ensure the stable averaging of noise. Ten participants (4 BPD, 1 SAD, 5 HC) had to be excluded due to an insufficient number of segments, leading to the sample of 85 participants described above. We were not able to consider the events “others” in the analysis because of the reduced number of EEG segments in the overinclusion condition (co-players rarely passed the ball to each other in this condition). We matched the number of segments for the event “self overinclusion” to the number of segments for the event “self inclusion” to obtain comparable signal-to-noise ratios.

Averages for each participant were calculated, separately for condition (inclusion, overinclusion), ball possession (self, others) and electrode position (frontal, central, parietal). Subsequently, grand averages were calculated for the three groups (HC, SAD, BPD) and the P3 time window (310–390 ms). The time window was determined based on the grand averages of the event-related potentials.

### 2.5 Statistical analysis

We used mixed ANOVAs to examine group differences in the processing of social inclusion and overinclusion. Dependent variables were a) need threat, b) ostracism intensity, c) estimated percentage of ball tosses received and d) P3 amplitudes. Independent variables were *group* (between-subject factor with 3 levels: HC, SAD, BPD) and *condition* (within-subject factor with 2 levels: inclusion, overinclusion). Significant effects of the ANOVA were further examined in Bonferroni-corrected post-hoc analyses. Pearson's *r* was used as a measure of effect size (small effect: *r* = 0.10; medium effect: *r* = 0.30; large effect: *r* = 0.50).

Analyses were conducted using R version 3.4.0 (R Core Team, 2014) and an alpha level of 0.05 was applied. To calculate the ANOVAs, we relied on a multi-level approach using the nlme package of R (Pinheiro et al., 2017). This enabled us to consider dependency in the data resulting from the repeated measurement (Field et al., 2012).

## 3 Results

Table 2 depicts means and standard deviations for all outcome variables as well as the results of the mixed ANOVAs.

**Table 2:** Means/SDs of the outcome variables and results of the mixed ANOVAs.

		HC	SAD	BPD	Mixed ANOVAs						
					Condition			Group		Condition × group	
		*M* (*SD*)	*M* (*SD*)	*M* (*SD*)	*χ*^2^(1)	*p*	*r*	*χ*^2^(2)	*p*	*χ*^2^(2)	*p*
P3 Pz	Inclusion	5.78 (3.53)	7.62 (3.19)	8.40 (4.39)	**41.03**	**<0.001**	**0.62**	**14.56**	**<0.001**	2.03	0.36
	Overinclusion	2.48 (2.79)	5.60 (3.17)	5.68 (3.47)							
Throws %^a^	Inclusion	31.25 (12.15)	29.43 (12.42)	26.61 (6.90)	**46.04**	**<0.001**	**0.65**	3.50	0.17	0.45	0.80
	Overinclusion	46.11 (16.98)	46.43 (20.92)	40.46 (13.09)							
Ostracism^b^	Inclusion	2.93 (1.36)	4.54 (2.47)	4.33 (2.17)	32.92	<0.001	0.59	6.64	0.04	**9.56**	**0.008**
	Overinclusion	2.39 (1.37)	2.32 (0.72)	2.48 (1.12)							
Neg. mood	Inclusion	1.20 (0.46)	1.93 (1.18)	1.81 (0.97)	1.90	0.17	0.16	8.22	0.02	**6.33**	**0.04**
	Overinclusion	1.30 (0.86)	1.38 (0.78)	1.78 (0.97)							
Need threat	Inclusion	9.33 (1.64)	12.7 (2.97)	12.5 (2.53)	26.91	<0.001	0.54	27.24	<0.001	**8.38**	**0.02**
	Overinclusion	8.3 (2.5)	9.07 (2.93)	10.55 (2.8)							

*Note*. HC = Healthy Controls, SAD = Social Anxiety Disorder, BPD = Borderline Personality Disorder. Throws % = Estimated Percentage of Ball Tosses Received, Neg. Mood = Negative Mood. Pz = parietal. Significant interaction effects are indicated in bold; if there was no significant interaction effect, significant main effects are indicated in bold instead.

a One person with BPD with missing data: n (BPD) = 28.

b Two persons with BPD with missing data: n (BPD) = 27.

### 3.1 EEG data: P3 complex

In a pre-analysis, we checked the effect of the electrode position on the P3 amplitude (*χ*^2^(2) = 199.58, *p* < 0.001). A contrast analysis revealed that, as expected, the P3 amplitude was more pronounced at the parietal compared to the frontal/central position (*t*(338) = 13.82 *p* < 0.001, *r* = 0.60). Means and *SDs* of the P3 amplitude at all electrode positions can be found in Table A.1 in the appendix.

**Fig. 2:**
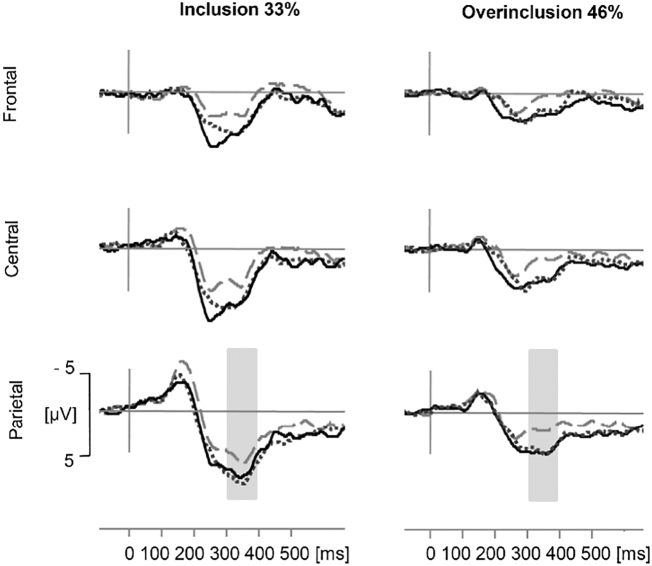
Grand averages of event-related potentials for both conditions (inclusion with 33% ball possession and overinclusion with 45% ball possession) and each group at three electrode positions (frontal, central, parietal). Dashed grey line = Healthy Controls, solid line = Borderline Personality Disorder, dotted line = Social Anxiety Disorder. Amplitude differences between the conditions and groups were examined for the P3 complex (310–390 ms) at parietal position.

Mean amplitudes of the parietal position indicated that the P3 was more pronounced in the inclusion compared to the overinclusion condition and that SAD patients and BPD patients showed a more pronounced P3 amplitude than HCs (see Fig. 2). Both main effects were confirmed in the statistical analysis (see Table 2). The interaction effect between condition and group on the P3 amplitude was not significant (see Table 2). Post-hoc analyses for the main effect of group revealed that the P3 was more pronounced in both clinical groups compared to HCs (HC vs. SAD: *p* = 0.009, *r* = 0.32; HC vs. BPD: *p* = 0.001, *r* = 0.37). The clinical groups did not differ in their P3 amplitude (SAD vs. BPD: *p* = 1, *r* = 0.06).

Fig. 2 depicts grand-averaged event-related potentials for each group at each position in both conditions.

### 3.2 Self-report data

The interaction effect of condition and group on ostracism intensity was significant (see Table 2). Bonferroni-corrected post-hoc analyses revealed that both clinical groups reported stronger feelings of ostracism than HCs in the inclusion condition (HC vs. SAD: *p* = 0.001, *r* = 0.38; HC vs. BPD: *p* = 0.006, *r* = 0.33). SAD patients and BPD patients did not differ in ostracism intensity in the inclusion condition (SAD vs. BPD: *p* = 1, *r* = 0.05). In the overinclusion condition, the three groups did not differ in their reported ostracism intensity (all *p* = 1).

The interaction effect of condition and group on negative mood was significant (see Table 2). The Bonferroni-corrected post-hoc analyses revealed that both clinical groups reported more negative mood than HCs in the inclusion condition, while the clinical groups did not differ (HC vs. SAD: *p* = 0.01, *r* = 0.32; HC vs. BPD: *p* = 0.04, *r* = 0.27; SAD vs. BPD: *p* = 1, *r* = 0.06). In the overinclusion condition, the groups did not differ in terms of negative mood (all *p* < 0.15).

The interaction effect of condition and group on need threat was significant (see Table 2). Post-hoc analyses revealed that both clinical groups reported higher need threat than HCs in the inclusion condition (HC vs. SAD: *p* < 0.001, *r* = 0.47; HC vs. BPD: *p* < 0.001, *r* = 0.45; SAD vs. BPD: *p* = 1, *r* = 0.03). In the overinclusion condition, only BPD patients reported higher need threat than HCs (HC vs. SAD: *p* = 0.80, *r* = 0.12; HC vs. BPD: *p* = 0.005, *r* = 0.34; SAD vs. BPD: *p* = 0.10, *r* = 0.23).

Participants estimated having received the ball more often in the overinclusion than in the inclusion condition (see Table 2). Thus, our experimental manipulation was successful. There was no significant effect of group and no significant interaction effect between group and condition on the estimated percentage of received ball tosses (all *p* > 0.17).

### 3.3 Further analysis of the inclusion condition

Finally, we examined whether we can replicate the results of Gutz et al. (2015) that patients with BPD process social inclusion in a biased manner. To test our hypothesis that the P3 complex would be enhanced in patients with BPD compared to HCs in the inclusion condition, we performed a one-way ANOVA with the independent variable *group* (3 levels: HC, SAD, BPD).

First, we checked the effect of the electrode position on the P3 amplitude in the inclusion condition (*χ*^2^(2) = 116.65, *p* < 0.001). As expected, the contrast analysis revealed that the P3 was more pronounced at the parietal compared to the frontal/central position (*t*(168) = 10.48, *p* < 0.001, *r* = 0.63).

In the inclusion condition, the P3 amplitude was more pronounced in BPD patients compared to HCs (see Table 2 for means and *SD*). The one-way ANOVA showed that group had a significant effect on the P3 amplitude in the inclusion condition (*χ*^2^(2) = 7.25, *p* = 0.03). Bonferroni-corrected post-hoc analyses revealed that the P3 amplitude was only significantly more pronounced in BPD patients compared to HCs (*p* = 0.03, *r* = 0.28). Differences in the P3 amplitude between SAD patients and HC and differences between SAD patients and BPD patients were not significant (all *p* > 0.21).

## 4 Discussion

The current study confirmed, with an EEG correlate, that individuals with BPD show a bias in the processing of social inclusion: Relative to healthy participants, individuals with BPD showed an enhanced P3 complex when included. Our results further imply that this bias is not specific to the situation of social inclusion: Even when overincluded, patients with BPD showed an enhanced P3 complex and felt a threat to their fundamental social needs relative to healthy participants.

The primary aim of this study was to examine whether patients with BPD show a biased processing only in the ambiguous situation of social inclusion or also when overincluded. When overincluded, BPD patients reported as much negative mood and ostracism as did HCs. However, the threat to social needs and the P3 amplitude were generally higher in BPD patients relative to HCs. These results are in line with the only previous study that looked at overinclusion in BPD (De Panfilis et al., 2015), which found that BPD patients experienced comparable levels of negative mood to HCs when overincluded, but felt less connected to the co-player irrespective of their current degree of social participation.

As part of the study, we replicated the finding of Gutz et al. (2015) that patients with BPD process social inclusion in a biased manner: When included, the P3 complex was enhanced in BPD patients compared to the non-clinical control group. This indicates that individuals with BPD expect to be excluded a priori, and social inclusion violates this expectation. Accordingly, this also specifies that the bias is already present in an initial stage of social information processing (Bartholow and Dickter, 2007). We also found evidence that patients with BPD *experience* (subjectively report) social inclusion in a biased manner. When included, patients with BPD reported more negative mood as well as ostracism and experienced more need threat compared to healthy participants.

It is necessary to specify our hypothesis that the bias in BPD is most prominent in the more ambiguous situation of social inclusion (Beck et al., 2015). On the one hand, our results imply that patients with BPD are able to recognize when they are extremely included, and consequently no longer feel excluded or sad/angry (which is in line with our hypothesis). On the other hand, in BPD, underlying constructs such as the need to belong might always be threatened in social interactions and social inclusion might always be unexpected (indicated by the enhanced P3 amplitude), irrespective of the current degree of social participation. This also fits with the finding of Gutz et al. (2015) that patients with BPD experienced more negative mood and ostracism than HCs only when included, but reported higher threat to their social needs when included *and* when excluded. Moreover, this finding corresponds with the negative thinking patterns in BPD (Roepke et al., 2012; Staebler et al., 2011a).

The generally enhanced P3 amplitude could be interpreted in the light of difficulties of BPD patients to adjust their prior expectations (e.g., “I will always be excluded”) to the current situation (e.g., being included). Hence, the P3 amplitude might be a possibility to measure the persistence of expectation, which seems to be a core feature of mental disorders (Rief et al., 2015). It should be noted that besides the violation of a priori social expectation, other mechanisms might have led to the generally enhanced P3 complex in BPD. For example, using a social feedback task, van der Veen et al. (2014) showed that the P3 amplitude is larger in response to positive outcomes. This is in line with studies linking the P3 amplitude to the motivational significance of stimuli (see Nieuwenhuis et al., 2005 for a review). Hence, the P3 amplitude might have been elevated in both patient groups, because social stimuli might be more significant to them. However, this cannot explain why the P3 amplitude is less increased in the overinclusion compared to the inclusion condition.

In contrast to the results of Gutz et al. (2015), all groups were quite accurate in their estimation of received ball tosses. This is in line with other studies reporting that BPD patients showed no difficulties to accurately estimate how often they received the ball (e.g., Domsalla et al., 2014). However, in some Cyberball studies, BPD patients generally underestimated how often they received the ball (Renneberg et al., 2012) or underestimated it only in the inclusion condition (Staebler et al., 2011b). Future research should target this heterogeneity of findings by identifying possible moderator variables (e.g., arousal, study design).

Patients with SAD reported more need threat, ostracism intensity and negative mood compared to HCs in the inclusion but not in the overinclusion condition. Moreover, patients with SAD showed a generally enhanced P3 amplitude compared to HCs. Thus, our results further imply that individuals with SAD show deviations in the processing of social participation as well. This extends previous findings that individuals high in social anxiety need longer to recover from social exclusion than individuals low in social anxiety (Heeren et al., 2017; Oaten et al., 2008); and that women high in social anxiety benefit less from social overinclusion than women low in social anxiety (Gilboa-Schechtman et al., 2014). However, when focusing on the inclusion condition (one-way ANOVA) differences between patients with SAD and HCs were non-significant. Thus, results have to be interpreted with caution. Moreover, in the inclusion condition, the P3 complex was not elevated in SAD compared to BPD patients. This contrasts with the results of Gutz et al. (2015). One possible explanation is that our SAD sample was more clinically impaired on the Social Phobia Inventory (Connor et al., 2000) than the SAD sample in the study by Gutz et al. (2015). Indeed, an exploratory analysis (see appendix Table A.2) revealed that our SAD sample had a significantly higher symptom load than SAD patients in the study by Gutz et al. (2015).

### 4.1 Limitations

Several limitations of the study design need to be mentioned: First, we had no exclusion condition and were thus only able to confirm the results of Gutz et al. (2015) for the inclusion condition. Second, we did not control for possible order effects (all participants were first included and then overincluded). However, in a previous study, the order of conditions had no effect on the P3 amplitude (Niedeggen et al., 2014). Third, in order to preserve the cover story, self-report questionnaires were applied after both conditions had been completed (and not directly after each condition). The slightly divergent results between self-report and EEG data might be explained by the different timing of assessments, as the EEG data were assessed continuously during the Cyberball game.

### 4.2 Conclusion

This is the first study to examine the processing of social inclusion and overinclusion in BPD and in SAD relying on EEG data. Our study replicated previous findings that individuals with BPD process and experience social inclusion in a biased manner. Moreover, we provided evidence that individuals with BPD are well able to recognize when they are extremely included, and consequently no longer feel ostracized. However, they seem to expect to be excluded and feel a threat to their social needs irrespective of their current degree of social participation.

In BPD, these deviations in the processing of social participation may decrease the probability of positive social interactions and may explain interpersonal problems of individuals with this disorder. These results have implications for clinical practice. Psychoeducation is needed to inform BPD patients about the possibility that they may feel excluded even though they were part of a group. BPD patients could be advised to behave in social situations as if they are included, even though they feel rejected (e.g., in group therapy). This might enable them to interrupt the vicious cycle of interpersonal problems related to perceived ostracism. Future research should target adaptive processes in social interactive situations that influence the modification of social expectation in individuals with BPD.

## Funding

No external funding was received for this study. We will apply for the Open Access Publication Fund of the Freie Universität Berlin.

## Figures and Tables

**Table A.1:** Means and SDs of the P3 amplitude at central, frontal and parietal position.

		HC (*n* = 28)	SAD (*n* = 28)	BPD (*n* = 29)
		*M* (*SD*)	*M* (*SD*)	*M* (*SD*)
P3 Fz	Inclusion	1.79 (2.94)	3.97 (4.13)	3.94 (3.57)
	Overinclusion	0.30 (2.14)	2.35 (3.18)	2.81 (2.34)
P3 Cz	Inclusion	3.93 (3.28)	6.42 (3.70)	6.41 (4.39)
	Overinclusion	1.23 (2.40)	4.05 (3.18)	4.18 (2.88)
P3 Pz	Inclusion	5.78 (3.53)	7.62 (3.19)	8.40 (4.39)
	Overinclusion	2.48 (2.79)	5.60 (3.17)	5.68 (3.47)

*Note*. HC = Healthy Controls, SAD = Social Anxiety Disorder, BPD = Borderline Personality Disorder. Fz = frontal, Cz = central, Pz = parietal.

**Table A.2:** Means and SDs of the SPIN scores.

	HC	SAD	BPD	*t*–Test for SAD groups	
	*M* (*SD*)	*M* (*SD*)	*M* (*SD*)	*t*(51)	*p*
SPIN (this study)	12.82 (10.99)	41.07 (10.99)	35.93 (13.48)	2.55	0.01
SPIN (Gutz et al., 2015)^a^	13.76 (7.10)	32.60 (13.16)	34.40 (12.87)		

*Note*. SPIN = Social Phobia Inventory; HC = Healthy Controls, SAD = Social Anxiety Disorder, BPD = Borderline Personality Disorder.

a *N* = 25 per group.
